# Prognostic impact of a lymphocyte activation-associated gene signature in GBM based on transcriptome analysis

**DOI:** 10.7717/peerj.12070

**Published:** 2021-08-25

**Authors:** Yujia Lan, Erjie Zhao, Xinxin Zhang, Xiaojing Zhu, Linyun Wan, Suru A, Yanyan Ping, Yihan Wang

**Affiliations:** Harbin Medical University, College of Bioinformatics Science and Technology, Harbin, China

**Keywords:** Lymphocyte activation-associated gene signature, Glioblastoma multiforme (GBM), Prognostic biomarker, Lymphocyte activity, Overall survival

## Abstract

**Background:**

Glioblastoma multiforme (GBM) is a highly, malignant tumor of the primary central nervous system. Patients diagnosed with this type of tumor have a poor prognosis. Lymphocyte activation plays important roles in the development of cancers and its therapeutic treatments.

**Objective:**

We sought to identify an efficient lymphocyte activation-associated gene signature that could predict the progression and prognosis of GBM.

**Methods:**

We used univariate Cox proportional hazards regression and stepwise regression algorithm to develop a lymphocyte activation-associated gene signature in the training dataset (TCGA, *n* = 525). Then, the signature was validated in two datasets, including GSE16011 (*n* = 150) and GSE13041 (*n* = 191) using the Kaplan Meier method. Univariate and multivariate Cox proportional hazards regression models were used to adjust for clinicopathological factors.

**Results:**

We identified a lymphocyte activation-associated gene signature (*TCF3*, *IGFBP2*, *TYRO3* and *NOD2*) in the training dataset and classified the patients into high-risk and low-risk groups with significant differences in overall survival (median survival 15.33 months vs 12.57 months, HR = 1.55, 95% CI [1.28–1.87], log-rank test *P* < 0.001). This signature showed similar prognostic values in the other two datasets. Further, univariate and multivariate Cox proportional hazards regression models analysis indicated that the signature was an independent prognostic factor for GBM patients. Moreover, we determined that there were differences in lymphocyte activity between the high- and low-risk groups of GBM patients among all datasets. Furthermore, the lymphocyte activation-associated gene signature could significantly predict the survival of patients with certain features, including IDH-wildtype patients and patients undergoing radiotherapy. In addition, the signature may also improve the prognostic power of age.

**Conclusions:**

In summary, our results suggested that the lymphocyte activation-associated gene signature is a promising factor for the survival of patients, which is helpful for the prognosis of GBM patients.

## Introduction

Glioblastoma multiforme (GBM) is classified as a grade IV diffuse glioma and is one of the most aggressive and lethal brain cancers. GBM has a high recurrence rate, typically originates in the cerebral hemispheres, and can quickly spread to the other parts of the brain (Alifieris & Trafalis, 2015; Batash, Asna & Schaffer, 2017). IDH status is a classical marker of GBM, and patients with IDH mutations tended to have a better survival (Reuss, Mamatjan & von Deimling, 2015; Burgenske, Yang & Sarkaria, 2019; Aldape, Zadeh & von Deimling, 2015). In previous studies, GBM patients can be divided into four distinct molecular subtypes based on gene expression profiling, including proneural, neural, classical and mesenchymal (Lee, Lee & Lee, 2018; Liang et al. , 2005). The standard treatment for GBM is surgery, followed by radiation and chemotherapy (Alifieris & Trafalis, 2015; Batash, Asna & Schaffer, 2017; Wick et al., 2018). Recently, immunotherapies have been introduced for the treatment of GBM, especially for patients with EGFR mutations. However, the median survival time for GBM patients is only 15 months, which suggests that drug treatments may be ineffective for most of patients (Zanders, Svensson & Bailey, 2019).

The lymphocyte activation is a set of processes where the lymphocytes are stimulated by specific antigens or nonspecific mitogens, which results in the synthesis of protein and production of lymphokines (Kay, 1991; Hodgkin, Chin & Hasbold, 1997). These processes affect the proliferation and differentiation of various effector and memory cells. The effector cells will respond to antigens for the first time during the primary immune response. The memory cells can respond to a secondary immune response, which is known as immunological memory. Lymphocyte activation is destroyed in cancer, which is important for immunotherapy (Burugu, Dancsok & Nielsen, 2018; Ware, 2008). Some lymphocyte activation-associated markers had been found to be associated with favorable survival and be effective for the treatments of patients, such as, lymphocyte activation gene 3 (LAG-3) (Wang, et al. , 2018; Saleh et al., 2019; He et al., 2016). In addition, the lymphocyte activation combining with radiotherapy may be a novel treatment regimen for cancer patients (Akiyoshi, Tanaka & Mori, 2019; Sato, Jeggo & Shibata, 2019). Therefore, it is critical to develop a lymphocyte activation-associated prognostic signature of GBM to improve the treatment of patients.

In our study, a lymphocyte activation-associated gene signature was developed, which predicted the overall survival of GBM patients in training dataset. And we validated its prognostic power in another two datasets. The signature was found to be an independent prognostic factor after adjusting for other clinicopathologic factors. Moreover, this signature not only predicted the survival of patients with IDH wild-type glioblastoma, but also patients after radiotherapy. These findings indicated the signature may serve as an effective prognostic biomarker for patients with GBM.

## Materials and Methods

### Datasets description

We downloaded three gene expression datasets (TCGA, GSE16011 (Gravendeel et al. , 2009) and GSE13041 (Lee, Scheck & Nelson, 2008)) from The Cancer Genome Atlas (TCGA) portal (https://portal.gdc.cancer.gov) and Gene Expression Omnibus (GEO) (Edgar, Domrachev & Lash, 2002; Barrett et al., 2013), which contained patient outcome and clinicopathological factors. The gene expression profile of each dataset was normalized by replacing the expression level e with log2(e+1). The patients without matched clinical information were excluded. Consequently, a total of 866 GBM patients (525 GBM patients from TCGA, 191 GBM patients from GSE13041 and 150 GBM patients from GSE16011) were included in this study. The TCGA dataset was treated as a training dataset, and another two datasets (GSE16011 and GSE13041) were used as independent validation cohorts. In the training dataset, the median survival time of GBM patients was 12.4 months (ranging from 0.1 to 127.5 months). And there were 144 classical, 155 mesenchymal, 83 neural, and 99 proneural GBM samples. In Table 1 we summarized clinicopathological data belonging to GBM patients in the TCGA dataset. The median survival of GBM patients was 13.0 months (ranging from 0.2 to 111.8 months) and 8.7 months (ranging from 0.2 to 150.7 months) in the validation datasets, respectively (Table 2).

**Table 1:** Clinical and pathological characteristics of GBM patients in TCGA.

Clinical features	Category	GBM, *n* = 525
Gender	Female	205
	Male	320
Age	Median (range)	59 (10–89)
Classical Subtype	Classical	144
	Mesenchymal	155
	Neural	83
	Proneural	99
Survival status	Alive	75
	Deceased	449
Follow-up from samples (months)	Median (range)	12.39 (0.1–127.5)

**Table 2:** Clinical and pathological characteristics of GBM patients in GSE13041 and GSE16011.

Clinical features	Category	GSE13041 (*n* = 191)	GSE16011 (*n* = 150)
Gender	Female	74	47
	Male	117	103
Age	Median (range)	54 (18–86)	55.44 (14.38–80.65)
Survival status	Alive	15	3
	Deceased	176	147
Follow-up from samples (months)	Median (range)	12.97(0.23–111.77)	8.7 (0.24–150.72)

### Identifying the lymphocyte activation-associated gene signature

Differential expression analysis was performed based on transcriptome profile of GBM patients by the “limma” R package in the training dataset. Genes with the cutoff criteria of —log2-fold change—≥1 and FDR < 0.000001 between tumor and normal tissues were regarded as differentially expressed genes (DEGs). Using the univariate Cox proportional hazards regression algorithm, we retained the DEGs that were strongly associated with the survival of patients with GBM (*P* < 0.05). We focused on the prognostic genes that participated in the lymphocyte activation process. The lymphocyte activation-related genes were collected by GO terms in Gene Ontology (Ashburner et al., 2000; Gene Ontology, 2021). Then, we further selected genes associated with overall survival by stepwise regression analysis in the training dataset. Ultimately, we kept genes with the smallest Akaike information criteria (AIC) value to construct the prognostic model. There were four genes (*TCF3*, *IGFBP2*, *TYRO3* and *NOD2*) that were identified in our study (Fig. S1). Finally, a risk score model was developed based on the gene expression weighted by regression coefficients of univariable Cox regression: Risk score = (−0.6246210 × expression level of *TCF3*) + (0.2992  × expression level of *IGFBP2*) + (0.2421068  × expression level of *TYRO3*) + (0.2155469  × expression level of *NOD2*). Based on this model, the patients were classified into the high- and low-risk groups by the median risk score in the training dataset.

### Statistical analysis

In the survival analysis, we analyzed only overall survival as the end point. The survival differences between the two groups were visualized by Kaplan–Meier analysis and were compared by the log-rank test. The prognostic value of the lymphocyte activation-associated gene signature was estimated by univariate and multivariate Cox proportional hazards regression models. The Cox proportional hazards regression model was used to calculate hazard ratio (HR) and 95% confidence intervals (CI). The *P*-values smaller than 0.05 were considered to be statistically significant. We obtained gene signatures (that is cell markers) of lymphocytes (including natural killer cells, T cells and B cells) from the CIBERSORT web portal (Newman et al., 2015) and MCP-counter web portal (Becht et al., 2016). We combined information from these two sources to get gene signatures of lymphocytes. The lymphocyte activity of each patient was calculated by single sample gene set enrichment analysis (ssGSEA) (Barbie et al., 2009) based on the gene signatures of lymphocytes. Each ssGSEA enrichment score represents the degree to which the genes in a particular gene set are coordinately regulated within a sample. In our study, the gene signature of lymphocytes means the gene set of ssGSEA and the lymphocyte activity means the enrichment score. The concordance index (C-index) was used to compare the prognostic efficacy among this signature, age, sex, and the combined model using a logistic regression with the aforementioned three variables, and the significant *p*-value were calculated by rcorrp.cens function in Hmisc package. R software (http://www.r-project.org) was used to perform all of the statistical analyses, version 3.5.1 (Packages: survival, survminer, limma, Hmisc, ggplot2, GSVA).

## Results

### Development of a prognostic signature related to lymphocyte activation

The gene expression profile and clinical information of GBM patients were obtained from TCGA, which were treated as the training dataset. There were 525 tumor samples and 10 normal samples. The tumor and normal samples came from different samples. We identified 2,364 differentially expressed genes between tumor and normal samples by using the limma package (FDR < 0.000001 and —log2-fold change— ≥1), including 933 up-regulated and 1,431 down-regulated genes (Fig. S2). We obtained 173 differentially expressed genes that could be used for prognosis by univariate Cox proportional hazards regression. Genes were subjected to the lymphocyte activation function, which was obtained from Gene Oncology (GO). This resulted in 12 lymphocyte activation-associated genes that were used for subsequent analysis. Finally, we obtained four genes (*TCF3*, *IGFBP2*, *TYRO3* and *NOD2*) that were significantly related to overall survival using a stepwise regression algorithm. We used the lymphocyte activation-associated gene signature to calculate each patient’s risk score based the four genes’ expression levels weighted by regression coefficients in the univariate Cox proportional hazards regression analysis (Fig. S1). Positive coefficients of *IGFBP2*, *TYRO3*, and *NOD2* (0.2992, 0.2421068 and 0.2155469) suggested that their expression was associated with poor prognosis in GBM patients, while the negative coefficient of *TCF3* (−0.6246210) indicated it was associated with better survival.

**Figure 1:**
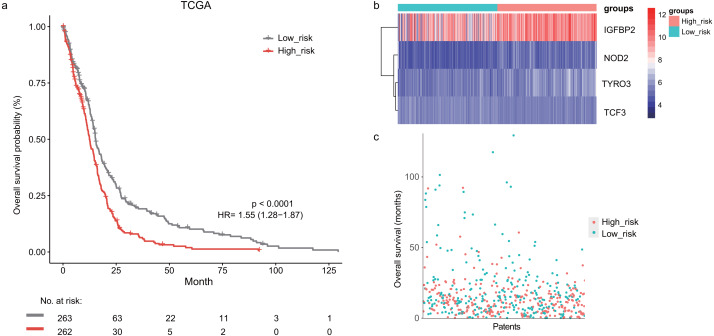
The survival analysis of the lymphocyte activation-associated gene signature in training set (TCGA, *n* = 525). (A) Kaplan–Meier curves of overall survival for the four gene signature. The patients were divided into high-risk (red) and low-risk group (grey). (B) The heatmap showing expression profiles of four genes in the signature. (C) The survival status and time distribution of the GBM patients.

In the training dataset, GBM patients were divided into high-risk (*n* = 262) and low-risk (*n* = 263) groups according to the median risk score (2.752) of the lymphocyte activation-associated gene signature. This score was used as the cutoff. Patients within high-risk group had significantly shorter overall survival time than those within low-risk group (median survival 15.3 months *vs* 12.6 months, HR = 1.55, 95% CI [1.28–1.87], log-rank test *P* < 0.001, Fig. 1A). The gene expression and survival time distributions suggested that patients in the high-risk group had higher *IGFBP2* expression (Fig. 1B). The low-risk patients tended to have a longer survival time (Fig. 1C).

### Prognostic value of the lymphocyte activation-associated gene signature in validation sets

**Figure 2:**
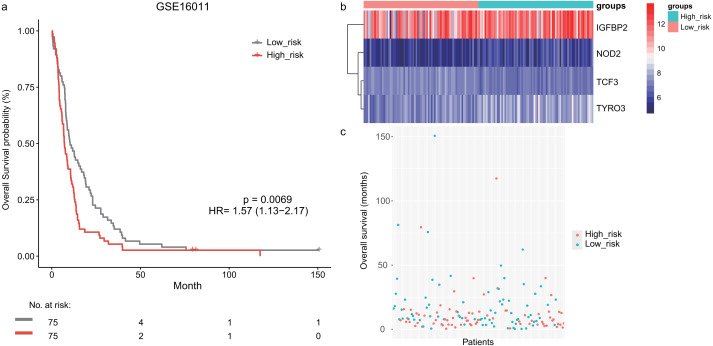
The survival analysis of the lymphocyte activation-associated gene signature in GSE16011 (*n* = 150). (A) Kaplan–Meier curves of overall survival for the four-gene signature with the median value as the cutoff. (B) The expression profiles of the four genes in the signature. (C) The distribution of the GBM patients’ overall survival status.

**Figure 3:**
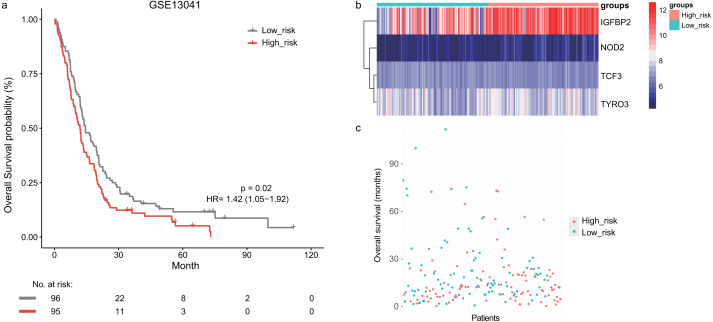
The survival analysis of the lymphocyte activation-associated gene signature in GSE13041 (*n* = 191). (A) Kaplan–Meier curves of overall survival for the four-gene signature with the median value as the cutoff. (B) The expression profiles of the four genes in the signature. (C) The distribution of the GBM patients’ overall survival status.

Next, we validated the prognostic value of the lymphocyte activation-associated gene signature in another two external validation datasets, GSE13041 (*n* = 191) and GSE16011 (*n* = 150). Each patient’s risk score was calculated with the same formula as that used in the training set. The patients were divided into the high- and low-risk groups according to the median cutoff determined for each dataset. In GSE16011, survival time of patients in the high-risk group were significantly shorter than those in the low-risk group (median survival 7.1 months *vs* 10.3 months, HR = 1.57, 95% CI [1.13–2.17], log-rank test *P* = 0.069, Fig. 2A). *IGFBP2* and *TYRO3* had a relatively higher expression in the high-risk group (Fig. 2B). We found the low-risk patients presented a trend of longer survival time in GSE16011 (Fig. 2C). Moreover, the signature was found to predict the survival of patients with GBM in GSE13041 (median survival of high-risk patients 12.0 months *vs* median survival of low-risk patients 14.5 months, HR = 1.42, 95% CI [1.05–1.92], log-rank test *P* = 0.02, Figs. 3A–3C) with statistical significance.

### The signature independently predicted overall survival of GBM patients

We used univariate and multivariable Cox regression analysis after adjusting for other clinicopathologic factors to assess whether the lymphocyte activation-associated gene signature was an independent prognostic biomarker in GBM. The covariables included age, sex, IDH status, subtypes, and the prognostic signature. We found that the signature (HR = 1.46, 95% CI [1.16–1.83], *P* = 0.001), age (HR = 1.03, 95% CI [1.02–1.04], *P* < 0.001), sex (HR = 1.30, 95% CI [1.04–1.63], *P* = 0.02) and proneural types (HR = 1.64, 95% CI [1.18–2.27], *P* = 0.003) independently predicted a worse OS for GBM patients in the training dataset (Table 3). In the validation dataset GSE16011, we also found that the lymphocyte activation-associated gene signature had independently prognostic value (HR = 1.36, 95% CI [1.35–1.98], *P* = 0.029, Table 4). This signature independently predicted the survival of GBM patients, with marginal significance (HR = 1.33, 95% CI [0.98–1.81], *P* = 0.06, Table S1). These findings revealed that the prognostic ability of the lymphocyte activation-associated gene signature was independent of the clinicopathological factors for OS in GBM.

**Table 3:** Multivariate analysis for the lymphocyte activation-associated gene signature of overall survival in TCGA.

Variables		Univariate			Multivariate		
		HR	95% CI	*P* value	HR	95% CI	*P* value
Age		1.032	1.025–1.04	<0.001^*^	1.028	1.018–1.037	<0.001^*^
Sex	Male *vs* Female	1.241	0.993–1.551	0.057	1.3	1.035–1.634	0.024^*^
IDH status	Mutation *vs* Wild type	0.353	0.229–0.544	<0.001^*^	0.831	0.396–1.742	0.623
Signature	High_risk *vs* Low_risk	1.588	1.275-1.979	<0.001^*^	1.455	1.159–1.828	0.001^*^
Subtypes	G-CIMP *vs* Classical	0.369	0.2271–0.599	<0.001^*^	0.701	0.319–1.544	0.378
	Mesenchymal *vs* Classical	1.169	0.876–1.56	0.289	1.117	0.835–1.494	0.455
	Neural *vs* Classical	1.091	0.788–1.509	0.6	1.177	0.848–1.633	0.331
	Proneural *vs* Classical	1.314	0.954–1.808	0.094	1.638	1.184–2.268	0.003^*^

**Notes.**

* Significant *P* values are labeled with * (*P* < 0.05).

**Table 4:** Multivariate analysis for lymphocyte activation-associated gene signature of overall survival in GSE16011.

Variables		Univariate			Multivariate		
		HR	95% CI	*P* value	HR	95% CI	*P* value
Age		1.039	1.024–1.054	<0.001^*^	1.031	1.024–1.039	<0.001^*^
Sex	Male *vs* Female	0.809	0.544–1.204	0.296	0.928	0.62-1.391	0.719
IDH status	Mutation *vs* wild type	0.579	0.367–0.915	0.019^*^	0.809	0.493-1.326	0.4
Signature	High_risk *vs* Low_risk	1.554	1.284–1.88	<0.001^*^	1.359	1.346–1.979	0.029^*^

**Notes.**

* Significant *P* values are labeled with * (*P* < 0.05).

### The differences in lymphocyte activity between the high- and low-risk groups of patients

**Figure 4:**
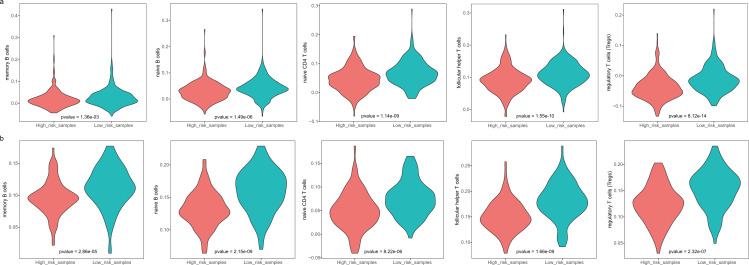
The cell activity of lymphocytes (memory B cells, native B cells, native CD4 T cells, follicular helper T cells and the regulatory T cells) in TCGA (A) and the GSE13041 (B) dataset.

The GBM patients were divided into low- and high-risk groups based on their lymphocyte activation-associated gene signature. We then compared the lymphocyte activity in these two groups. Lymphocytes include natural killer cells, T cells and B cells. In TCGA, five types of lymphocytes showed significant differences in activity between high- and low-risk patients by Wilcoxon ranked sum test (*P* < 0.05). These lymphocytes were memory B cells, naive B cells, naive CD4 T cells, follicular helper T cells and regulatory T cells (Fig. 4A and Fig. S3). And the lymphocyte activity of high-risk patients was significantly lower than in low-risk patients. Similar phenomena were observed in the GSE13041 dataset (Fig. 4B). However, activated CD4 memory T cells, resting CD4 memory T cells, CD8 T cells and gamma delta T cells showed significant differences between the two groups. There were three types of cells that showed different activity between low- and high-risk patients in the GSE16011 dataset (*P* < 0.05, Fig. S4). These findings suggested that lymphocyte activity was significantly different between the high- and low-risk GBM patients.

### Stratification analysis of the lymphocyte activation-associated gene signature

**Figure 5:**
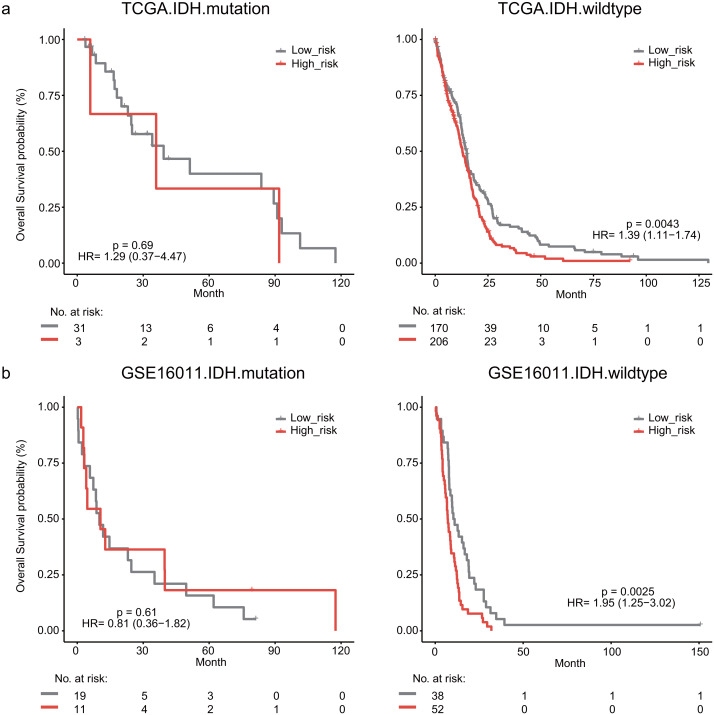
Kaplan–Meier analysis estimates of overall survival in TCGA (A) and the GSE16011 (B) datasets according to the IDH1 mutation status.

The IDH mutation is one of the most critical genomic alterations in GBM. The IDH mutation means a somatic mutation in IDH1 in our study. IDH-wildtype GBM patients had a shorter survival time than those with the IDH-mutation. The lymphocyte activation-associated gene signature significantly predicted the overall survival of IDH-wildtype GBM patients by log-rank test (*P* = 0.0043) but it did not predict the survival of IDH-mutation patients (Fig. 5A). The IDH-wildtype patients were divided into high-risk and low-risk groups using the same cutoff in the training dataset. The high-risk group of IDH-wildtype patients had significantly shorter OS than those in the low-risk group (median survival 12.9 months *vs* 14.9 months, HR = 1.39, 95% CI [1.11–1.74], *P* = 0.0043, Fig. 5A). The same phenomenon was also found in the GSE16011 dataset. The high-risk patients showed worse OS than the low-risk patients (median survival 7.1 months *vs* 10.7 months, HR = 1.95, 95% CI [1.25–3.02], *P* = 0.0025) among IDH-wildtype patients (Fig. 5B).

**Figure 6:**
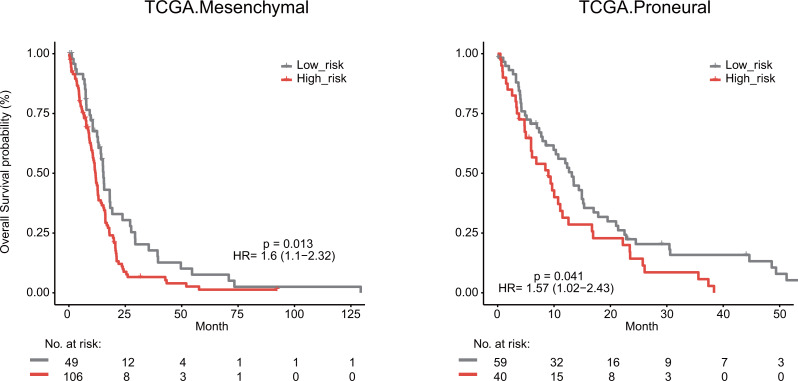
Survival analysis for GBM patients according to TCGA mesenchymal and proneural subtypes.

We next explored whether the lymphocyte activation-associated gene signature was effective for GBM patients within four transcriptome subtypes (proneural, neural, classical, and mesenchymal) established by Verhaak et al. using Kaplan–Meier survival analysis. The signature significantly predicted the overall survival of patients with the mesenchymal and proneural subtypes (log-rank test *P* value = 0.013 for mesenchymal subtypes and *P* value = 0.041 for proneural subtypes, Fig. 6). The patients in the high-risk groups had a shorter survival time than those in the low-risk groups (median survival 11.8 months *vs* 15.3 months for mesenchymal subtypes and 9 months *vs* 13.2 months for proneural subtypes, respectively). In addition, the signature did not have the prognostic ability for patients with neural and classical subtypes (Fig. S5).

**Figure 7:**
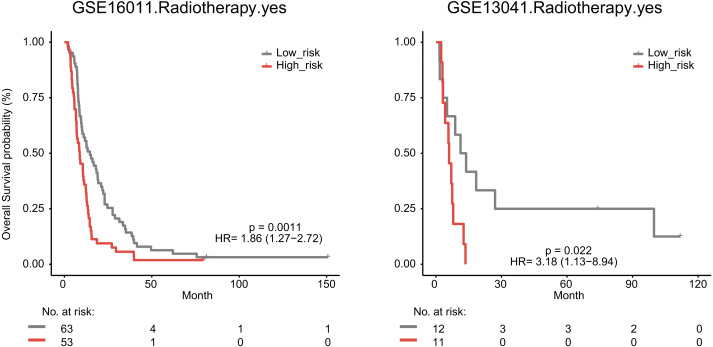
The signature predicts cancer patient outcome after radiotherapy.

We also explored whether radiotherapy was an effective treatment for GBM patients. We observed that high risk score significantly predicted a poor OS for GBM patients undergoing radiotherapy in the GSE16011 (median survival 8.76 months *vs* 14.64 months, HR = 1.86, 95% CI [1.27–2.72], *P* = 0.0011) and GSE13041 datasets (median survival 6.03 months *vs* 12.53 months, HR = 3.18, 95% CI [1.13–8.94], *P* = 0.022, Fig. 7).

### Comparison of prognostic power between clinical factors and the signature

To evaluate the prognostic performance of the prognostic signature, we performed C-index calculation of clinical factors (age and sex) and the signature in three datasets (Harrell Jr et al, 1982; Pencina & D’Agostino, 2004). We constructed another prognostic model by combining our signature with clinical factors. A higher C-index indicated a superior prognostic value of signature (Wu, Yuan & Liu, 2019; Li, Cui & Li, 2017). There were significant differences found between age and the signature (*P* < 0.05) and between age and sex. The clinical factors for age in combination with the signature showed a higher C-index (0.646 for TCGA, 0.665 for GSE16011 and 0.600 for GSE13041) than age or histological grade alone (0.645 for TCGA, 0.659 for GSE16011 and 0.595 for GSE13041, Table S2), which were statistically significant (*P* < 0.05). These results suggested that this lymphocyte activation-associated gene signature could add complementary value to known clinical factors.

## Discussion

In this study, we identified a lymphocyte activation-associated gene signature that predicted shorter overall survival of patients with GBM. The prognostic power of this signature was evaluated by univariate and multivariate Cox proportional hazards regression models analysis using three datasets (HR > 1). Our findings indicated that the gene signature was an unfavorable factor in GBM. Moreover, the high-risk and low-risk patients, which were separated by the signature, showed significant differences in immune cell activity. This signature was found to be an independent prognostic factor after adjusting for certain clinicopathological factors. In addition, the lymphocyte activation-associated gene signature also predicted the survival of patients with an IDH wild-type variant and for patients after radiotherapy treatment.

We developed a prognostic signature that included four genes (*TCF3*, *IGFBP2*, *TYRO3* and *NOD2*). *IGFBP2* was one of the insulin-like growth factor binding proteins (IGFBPs), which are proteins binding to Insulin-like growth factors. *IGFBP2* expression increased in peripheral blood mononuclear cells and participated in lymphocyte proliferation (Hettmer et al., 2005). In various autoimmune diseases, *IGFBP2* can be as potential biomarker and therapeutic target (Ding & Wu, 2018). Moreover, *IGFBP2* expression predicted the survival in GBM patients (Yuan et al., 2019; Cai et al., 2018). *NOD2* played important roles in the pathogenesis of some diseases, such as, oral lichen planus (Ahn et al., 2020), Crohn’s disease (Niess et al., 2012), and inflammatory bowel disease (Franchi et al., 2008). *NOD2* expression is higher in activated/memory CD4+ T cells (Zanello et al., 2013) and was found to be a new diagnostic and treatment target for disease (Ahn et al., 2020). A prognostic model was constructed by 9 immune genes, including NOD2, which predicted the shorter survival of GBM patients (Liang, Chai & Wang, 2020).

Another marker, an important transcription factor *TCF3* played a role in germinal center B - cell development and promoted cell growth, which contributed to proliferative phenotype in Burkitt lymphoma (Richter et al., 2012; Dave et al., 2006; Sakata-Yanagimoto et al., 2014). In addition, *TCF3* can regulate B-cell-restricted genes through E-box motifs (Barbie et al., 2009). It can promote survival of Burkitt’s lymphoma cells by activating B-cell receptor signaling and PI3K signaling pathways and by modulating cell cycle regulators (Schmitz et al., 2012). Also, *TCF3* promotes the survival in lymphoid cells (Sakata-Yanagimoto et al., 2014). *TYRO3* is a protein-coding gene, which participates in ERK signaling pathway. The higher level of *TYRO3* expression is associated with decreased overall survival in patients with colorectal, hepatocellular, and breast cancers (Smart et al., 2018). Although the latter two genes have not been directly shown to be associated with GBM, the prognostic efficacy of these two genes should be verified in future studies.

In conclusion, we developed a lymphocyte activation-associated gene signature with prognostic power and offered new insights for the treatments of GBM. However, more data are needed to test the prognostic value of the signature before applying it to clinical management. A larger study is needed to confirm that the signature can accurately predict the prognostic benefits for GBM patients.

##  Supplemental Information

10.7717/peerj.12070/supp-1 Supplemental Information 1 Supplemental Figures and Tables

## Additional Information and Declarations

### Data Availability

The following information was supplied regarding data availability: The data are available at TCGA (TCGA-GBM) and NCBI GEO (GSE16011 and GSE13041).
