# Supplementary material for: Prognostic impact of a lymphocyte activation-associated gene signature in GBM based on transcriptome analysis
Source: PeerJ. 2021 Aug 25;9:e12070. doi: 10.7717/peerj.12070 (PMC8401751; doi:10.7717/peerj.12070)
Supplement: Supplemental Information 1 [file peerj-09-12070-s001.doc]

**SUPPLEMENTARY INFORMATION**

**Supplementary Figures**

**
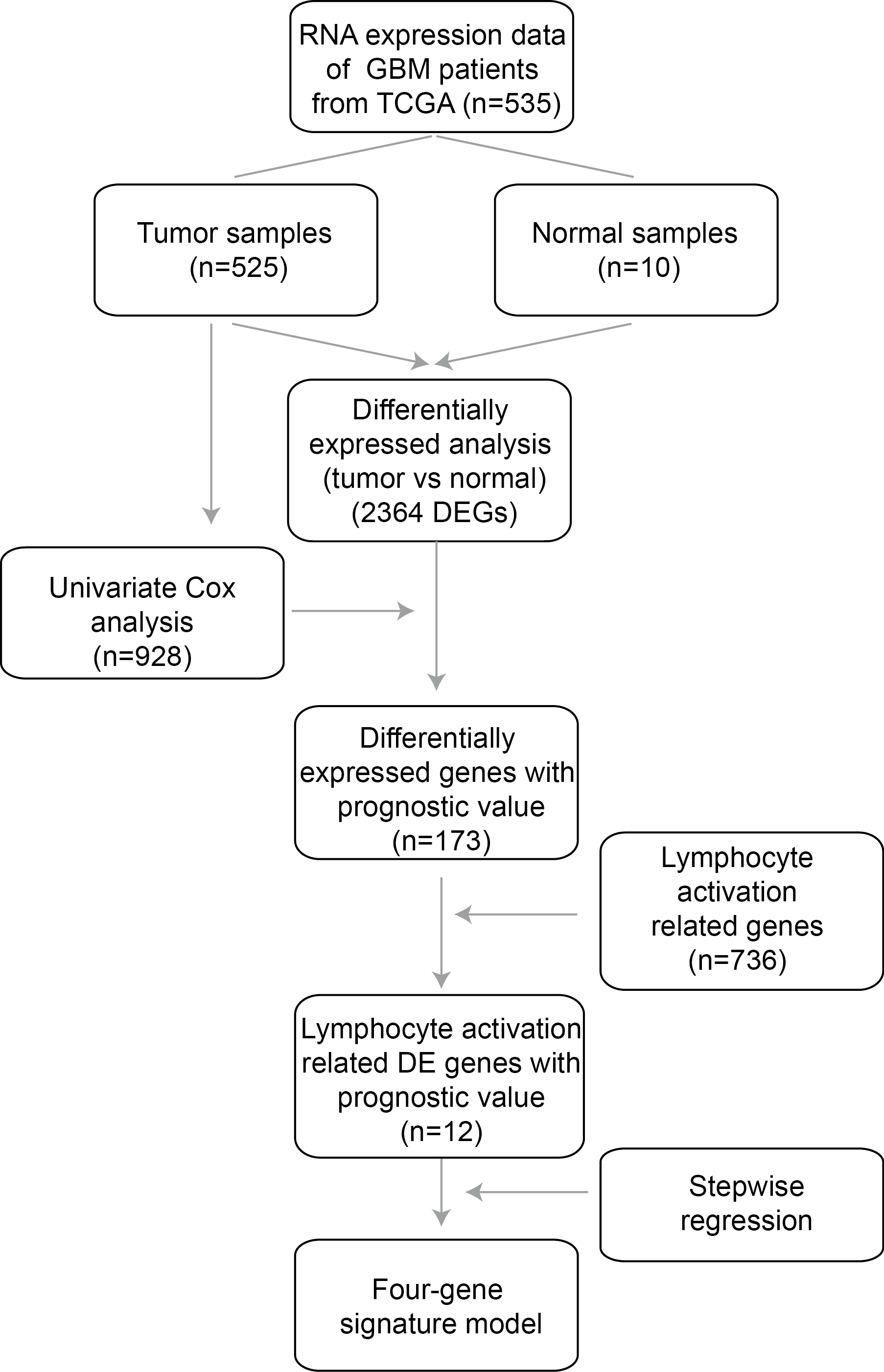
**

Supplementary Figure 1. The workflow of identification of the lymphocyte activation-associated gene signature.

**
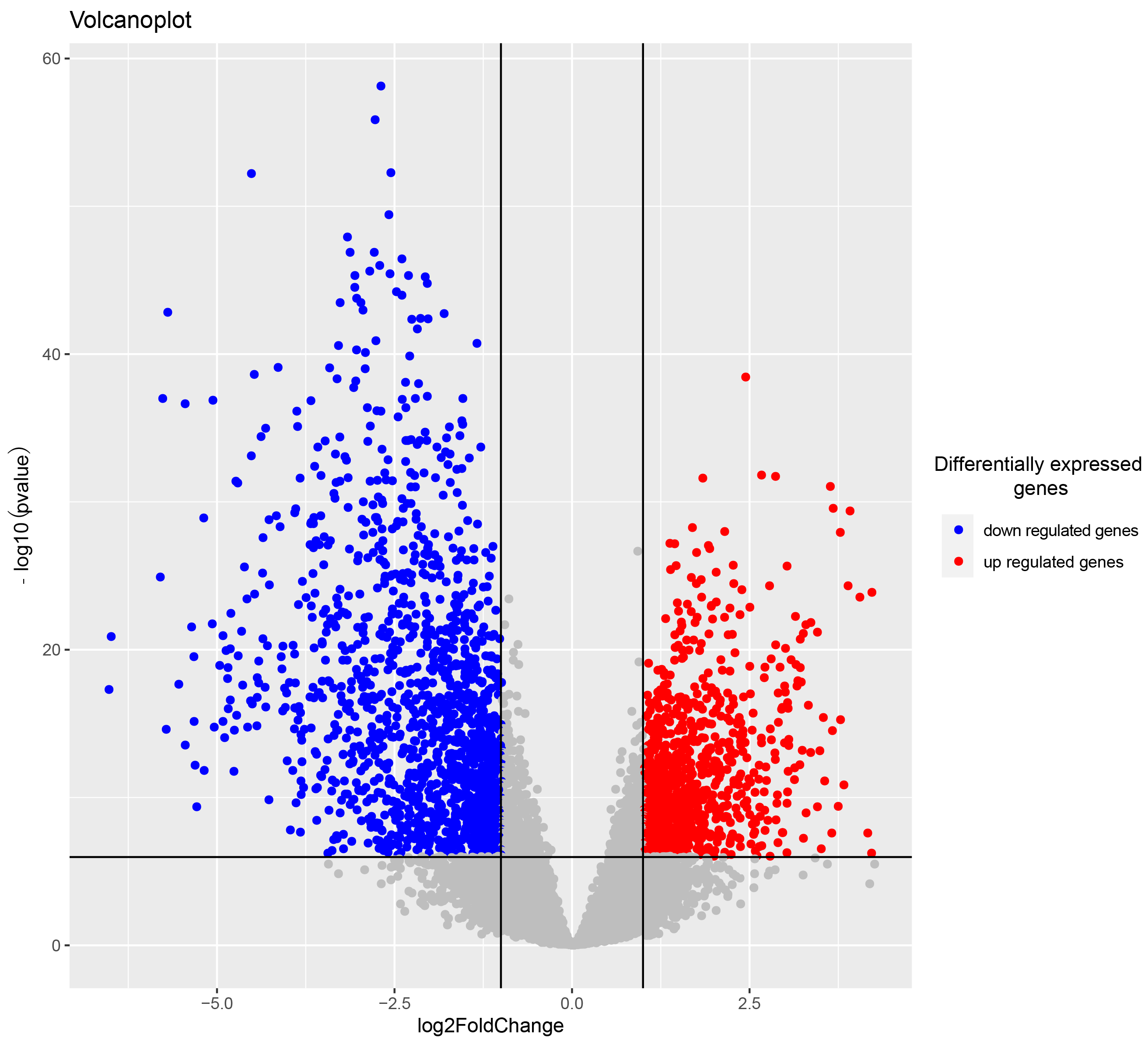
**

Supplementary Figure 2. The volcano plot of differentially expressed genes between cancer and normal cases in the TCGA dataset.

**
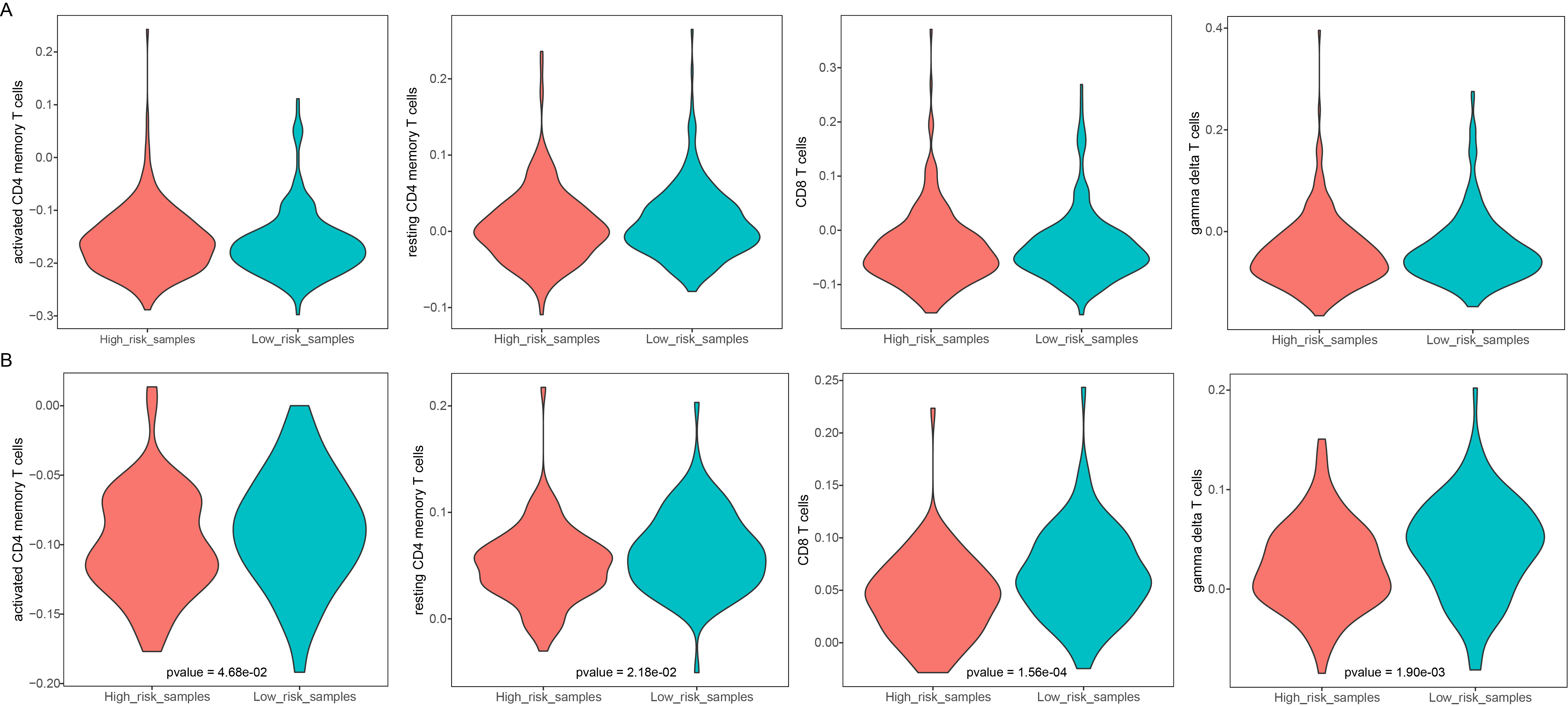
**

Supplementary Figure 3. The cell activity of lymphocytes (activated CD4 memory T cells, resting CD4 memory T cells, CD8 T cells and gamma delta T cells) in TCGA (A) and GSE13041 (B) data set.


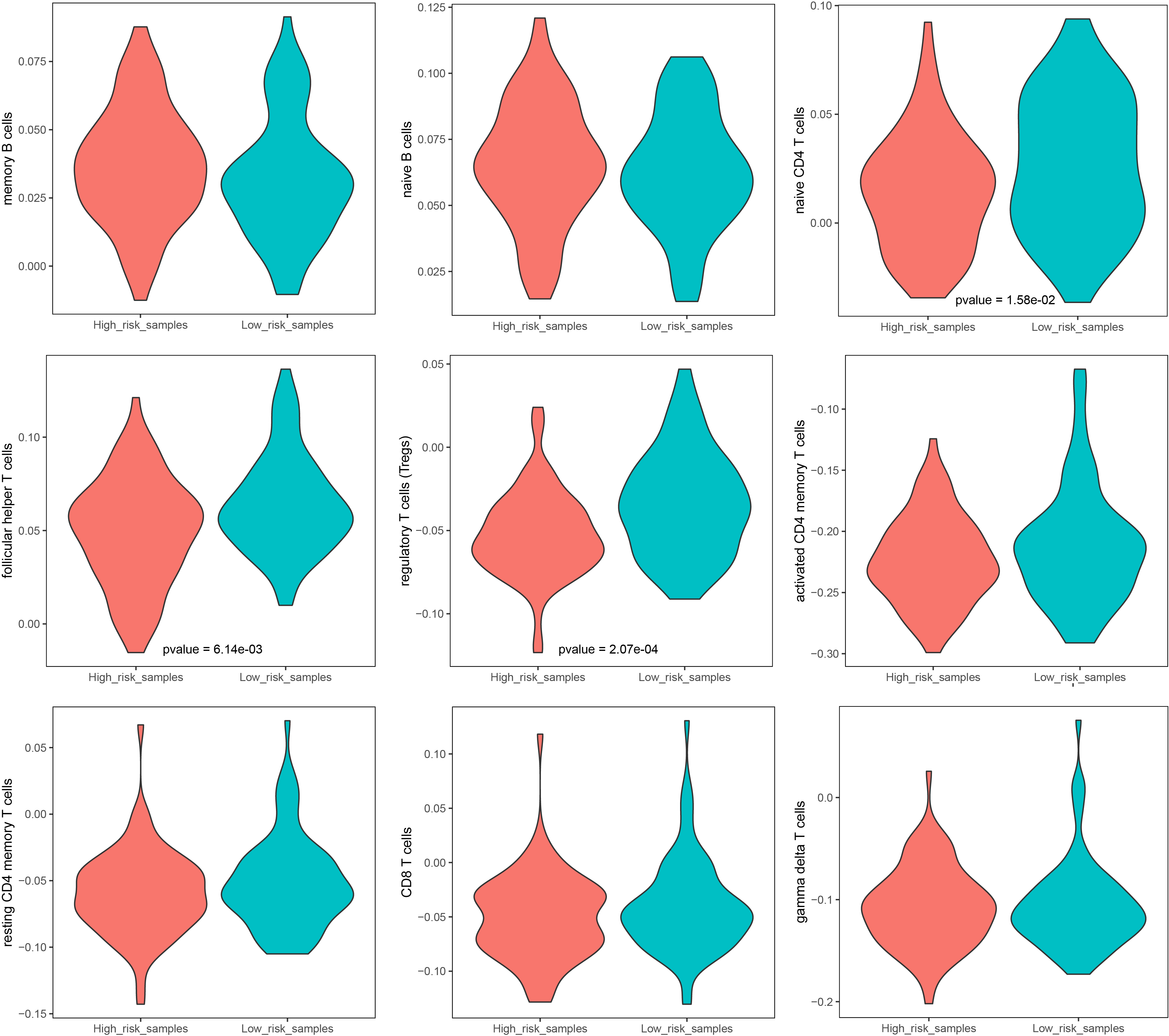


Supplementary Figure 4. The lymphocyte activity in GSE16011 data set.


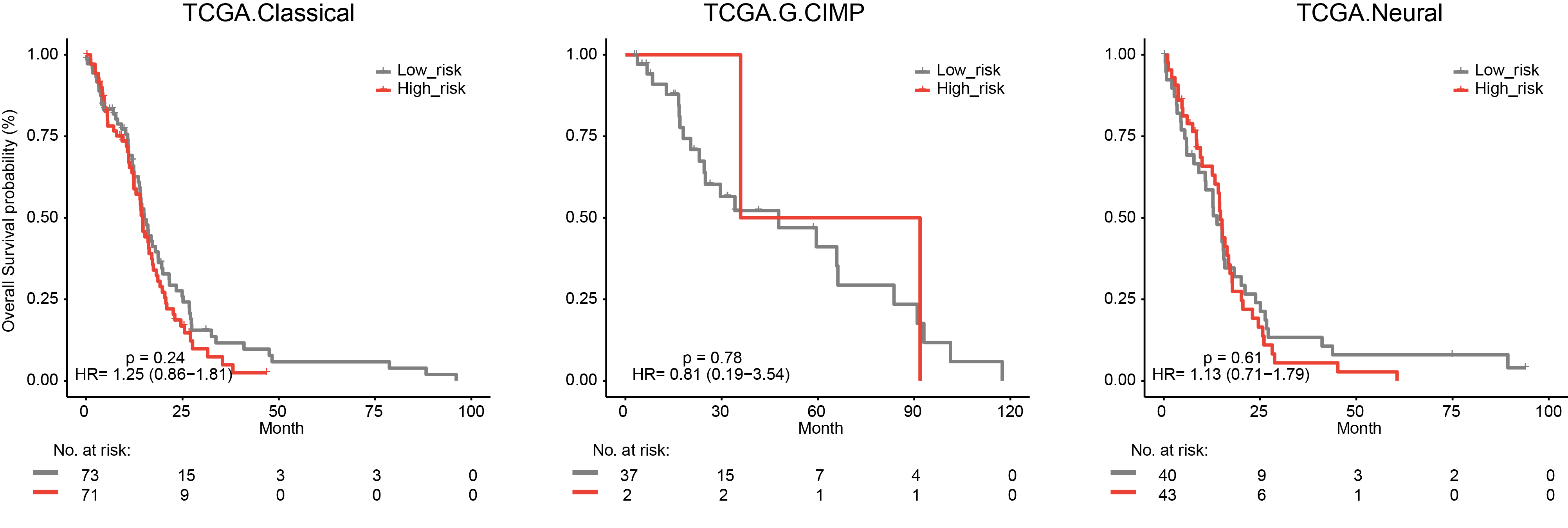


Supplementary Figure 5. Survival analysis for GBM patients according to TCGA neural and classical subtypes.

**Supplementary Tables**

Supplementary Table 1 Multivariate analysis for the lymphocyte activation-associated gene signature of overall survival in GSE13041.

| Variables |  |  | Univariate |  |  |  | Multivariate |  |
| --- | --- | --- | --- | --- | --- | --- | --- | --- |
|  |  | HR | 95% CI | p value |  | HR | 95% CI | p value |
| Age |  | 1.025 | 1.013-1.037 | <0.001^*^ |  | 1.024 | 1.012-1.036 | <0.001^*^ |
| Sex | Male vs Female | 0.961 | 0.708-1.303 | 0.796 |  | 0.947 | 0.695-1.291 | 0.731 |
| Signature | High_risk vs Low_risk | 1.421 | 1.053-1.916 | 0.021^*^ |  | 1.33 | 0.98-1.805 | 0.067 |

Significant *P* values are labeled with * (*P* < 0.05).

Supplementary Table 2. The C-index between clinical factors and the signature in three data sets.

|  |  | C-index | 95% CI | p value |
| --- | --- | --- | --- | --- |
| TCGA |  |  |  |  |
|  | Age | 0.645 | 0.615-0.675 | - |
|  | Signature | 0.547 | 0.520-0.573 | <0.001 |
|  | Sex | 0.511 | 0.484-0.537 | <0.001 |
|  | Combination | 0.646 | 0.616-0.675 | 0.040 |
| GSE16011 |  |  |  |  |
|  | Age | 0.659 | 0.607-0.712 | - |
|  | Signature | 0.564 | 0.518-0.610 | <0.001 |
|  | Sex | 0.510 | 0.467-0.552 | <0.001 |
|  | Combination | 0.665 | 0.613-0.717 | 0.129 |
| GSE13041 |  |  |  |  |
|  | Age | 0.595 | 0.546-0.645 | - |
|  | Signature | 0.541 | 0.499-0.582 | 0.033 |
|  | Sex | 0.510 | 0.469-0.551 | <0.001 |
|  | Combination | 0.600 | 0.549-0.649 | 0.050 |
